# Improving the catalytic characteristics of phenolic acid decarboxylase from *Bacillus amyloliquefaciens* by the engineering of N-terminus and C-terminus

**DOI:** 10.1186/s12896-021-00705-7

**Published:** 2021-07-26

**Authors:** Qin Li, Ying Xia, Ting Zhao, Yuanyuan Gong, Shangling Fang, Maobin Chen

**Affiliations:** 1 Key Laboratory of Fermentation Engineering (Ministry of Education), Hubei Provincial Cooperative Innovation Center of Industrial Fermentation, Hubei Key Laboratory of Industrial Microbiology, School of Food and Biological Engineering, Hubei University of Technology, No.28, Nanli Road, Wuhan, 430068 China; 2 Sichuan Agricultural University, No. 46, Xinkang Road, Yaan, 625014 China

**Keywords:** Phenolic acid decarboxylase, N-terminus, C-terminus

## Abstract

**Background:**

4-vinylphenols produced by phenolic acid degradation catalyzed by phenolic acid decarboxylase can be used in food additives as well as flavor and fragrance industry. Improving the catalytic characters of phenolic acid decarboxylase is of great significance to enhance its practical application.

**Results:**

A phenolic acid decarboxylase (P-WT) was created from *Bacillus amyloliquefaciens* ZJH-01. Mutants such as P-C, P-N, P-m1, P-m2, P-Nm1, and P-Nm2 were constructed by site-directed mutagenesis of P-WT. P-C showed better substrate affinities and higher turnover rates than P-WT for p-coumaric acid, ferulic acid, and sinapic acid; however, P-N had reduced affinity toward p-coumaric acid. The extension of the C-terminus increased its acid resistance, whereas the extension of the N-terminus contributed to the alkali resistance and heat resistance. The affinity of P-m1 to four substrates and that of P-m2 to p-coumaric acid and ferulic acid were greatly improved. However, the affinity of P-Nm2 to four phenolic acids was greatly reduced. The residual enzyme activities of P-Nm1 and P-Nm2 considerably improved compared with those of P-m1 and P-m2 after incubation at 50 °C for 60 min.

**Conclusions:**

The extension of the N-terminus may be more conducive to the combination of the binding cavity with the substrate in an alkaline environment and may make its structure more stable.

**Supplementary Information:**

The online version contains supplementary material available at 10.1186/s12896-021-00705-7.

## Background

**Fig. 1:**
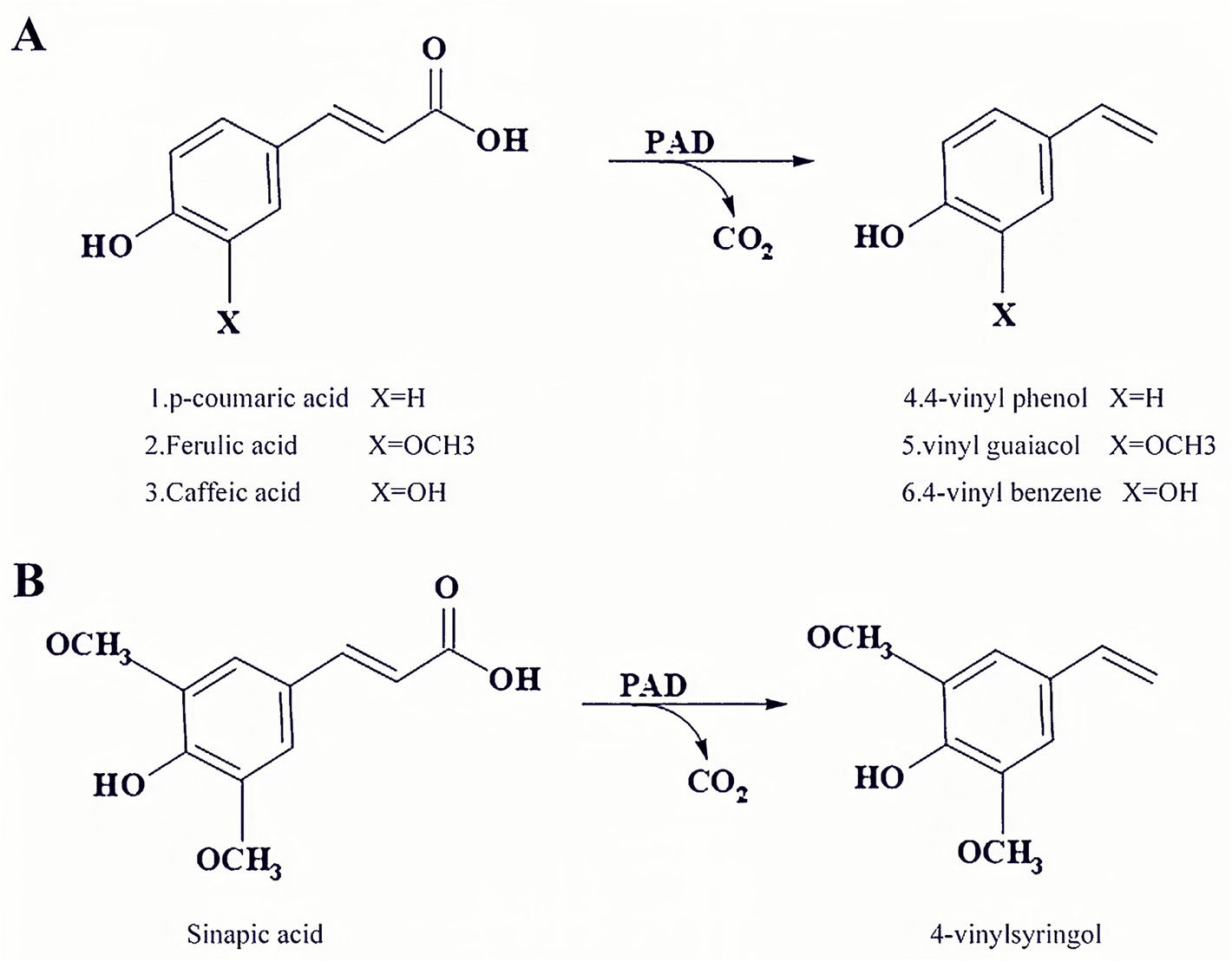
Phenolic acid decarboxylase catalyzes reactions of different substrates. **A** p-coumaric acid, ferulic acid, and caffeic acid. **B** sinapic acid

Phenolic acid is a general term for a class of organic acids containing phenolic rings, which exist in plants and can resist pathogens [1, 2]. Phenolic acids mainly include benzoic and cinnamic acid derivatives. Among them, benzoic acid derivatives primarily include gallic acid and protocatechuic acid, and cinnamic acid derivatives mainly include p-coumaric acid (PCA), ferulic acid (FA), caffeic acid (CA), and sinapic acid (SA) [3]. Phenolic acids are important plant compounds involved in the formation of molecular bonds among cellulose, hemicellulose, and lignin in cell walls, which are essential in the diet of mammals because their specific structure endows phenolic acids with important biological activities, making it possible to scavenge free radicals [4]. Some bacteria have a detoxification system mediated by the phenolic acid decarboxylase enzyme, which can synthesize phenolic acid, release carbon dioxide, and produce less toxic compounds called vinyl derivatives (Fig. 1) [5]. 4-vinyl guaiacol (3-methoxy-4-hydroxystyrene) and 4-vinyl phenol (4-hydroxystyrene) are 4-vinyl derivatives of phenolic acids characterized by unique odor and volatility, these can be used in food additives as well as flavor and fragrance industry [6]. Most of the 4-vinyl derivatives available in the market are chemically synthesized, requiring more precursor materials and more stringent conditions, such as microwave heating, high temperature, and high pressure, which pose considerable unknown hazards in the food industry [7]. It is feasible to produce 4-vinyl derivatives through biosynthesis because it is a relatively cheap and renewable natural raw material.

Since Zago et al. [8] cloned the phenolic acid decarboxylase from *Bacillus pumilus* and successfully expressed the gene in *Escherichia coli* and proved its decarboxylation effect on FA, PAD genes were cloned from several strains, including *Lactobacillus plantarum* [9], *Bacillus subtilis* [10], and *Enterobacter* [11] corresponding genes, and research on a series of enzymatic properties was conducted [12]. The great interest in phenolic acid decarboxylase encouraged researchers to conduct several structural and mechanistic studies on this enzyme for establishing the molecular determinants of its substrate selectivity and mechanism [11]. However, reports about whether the catalytic characteristic including substrate specificity could be tuned by altering the N/C-terminal region of phenolic acid decarboxylase were limited [8, 11, 13, 14].

In our previous work, the gene of phenolic acid decarboxylase (P-WT) from *Bacillus amyloliquefaciens* ZJH-01 was expressed in the *E. coli* BL21 (DE3) [15]. The protein model referenced cited the UniProt entry with PDB in this article was 2W2A(https://www.uniprot.org/uniprot/?query=2W2A&sort=score). This entry corresponds to phenolic acid decarboxylase from *Lactobacillus plantarum* (strain ATCC BAA-793/NCIMB 8826/WCFS1). Viewing the 3D structure (2W2A.pdb), it may be seen that each monomer has two beta-sheets and the two cores correspond to these two beta-sheets. The mutations in this article were only for monomers. The main differences between P-WT and phenolic acid decarboxylases from other bacteria were C-terminal and N-terminal extension [16] and some sites in the substrate binding cavity showed a certain degree of variation, especially E99, L129, and V131. These residues played an important role in the substrate specificity of PAD. A detailed analysis of the molecular interaction between the substrate binding cavity and *trans-p-coumaric* acid in these structures shows that the substrate can be stabilized by two hydrogen bonds with residues E99 and W24 when E–L–V is present [17]. So we created six hybrid enzyme by replacing the C-terminal extension (P-C) (the name of the mutant in bracket) and N-terminal extension (P-N) of P-WT with that of chain A of *Lactobacillus plantarum* (PDB code: 2W2A) [9], and site-directed mutagenesis of His92Glu (P-m1), Tyr122Leu (P-m2) and combination the N-terminal replacement with His92Glu (P-Nm1), Tyr(122) Leu (P-Nm2).

## Results and discussion

### Production of P-WT and mutants

**Fig. 2:**
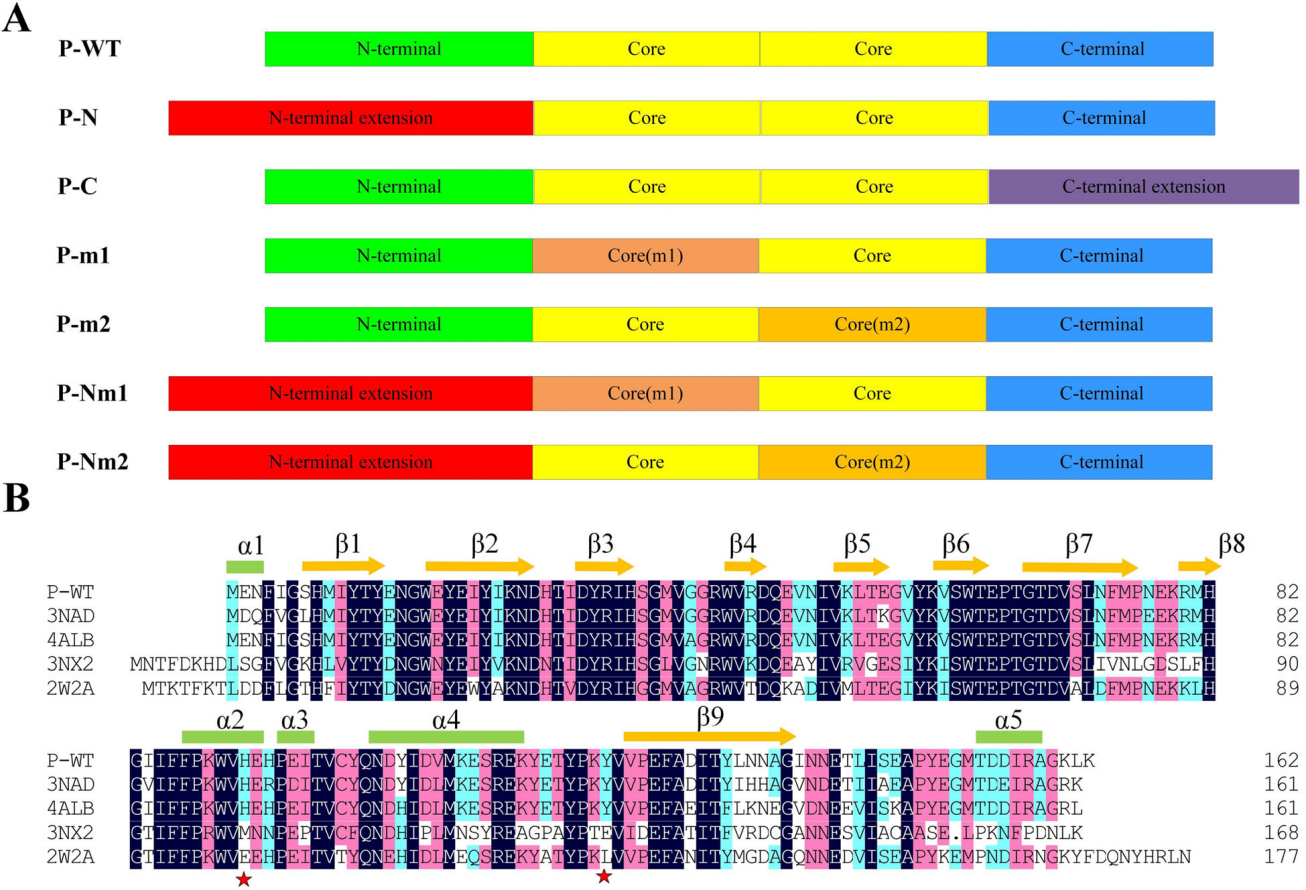
The formation of mutants for a monomer. **A** Schematic overview of the recombinant derivatives of P-WT. P-WT is the phenolic acid decarboxylase isolated from *Bacillus amyloliquefaciens* ZJH-01. P-N extended the N-terminal of P-WT. P-C extended the C-terminus of P-WT. P-m1 mutated the His92 to 92Glu. P-m2 mutated the Tyr122 to 122Leu. P-Nm1 extended the N-terminus of P-WT and mutated the His92 to 92Glu. P-Nm2 extended the N-terminus of P-WT and mutated the Tyr122 to 122Leu. We firstly measured the enzyme activity and specific activity of the N-terminal and C-terminal mutants, and found that the N-terminal had a greater influence on it than the C-terminal. So m1 and m2 combined with N-terminal but not with C-terminal. **B** The alignment of the amino acid sequence of the phenolic acid decarboxylase enzyme isolated from different strains. The P-WT is a phenolic acid decarboxylase in this experiment. The 3NAD, 2W2A, 3NX2 were obtained from the protein database

**Fig. 3:**
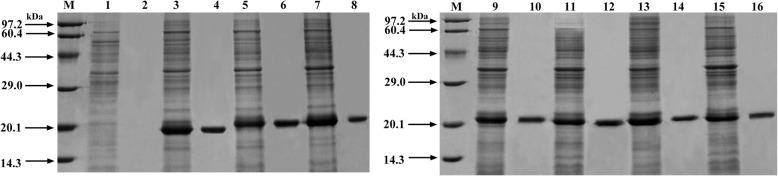
Analysis of P-WT and mutants by SDS-PAGE. M: Protein Marker; 1: Blank control with the empty vector; 2: Purified blank control with the empty vector; 3: P-WT; 4: Purified P-WT. 5: P-m1; 6: Purified P-m1; 7: P-m2; 8: Purified P-m2; 9: P-C; 10: Purified P-C; 11: P-N; 12: Purified P-N; 13: P-Nm1; 14: Purified P-Nm1; 15: P-Nm2; 16: Purified P-Nm2

The gene in this study was derived from *Bacillus amyloliquefaciens* ZJH-01 (P-WT). The open reading frame of the structural gene was 489 bp, which encoded 162 amino acids with a theoretical molecular weight of 19.2 kDa. The formation of each mutant was displayed in Fig. 2A. P-N, the N-terminus extension of P-WT, was composed of 12 amino acids (1Met-12Leu). P-C, the C-terminus extension of P-WT, was composed of 14 amino acids (157Asn-170Lys). The mutation positions of P-m1 and P-m2 were shown in Fig. 2B. P-m1, His92 of P-WT, was replaced with Glu92. P-m2, Tyr122 of P-WT, was replaced with Leu122. P-Nm1 was extended to the N-terminus of P-WT as well as mutated the His92 to 92Glu. P-Nm2 was extended to the N-terminal of P-WT and mutated Tyr122 to 122Leu. The collected enzymes were subjected to crushing, centrifugation, and dialysis, and the crude enzyme was purified by Ni Sepharose HP column. The purification results of P-WT and mutants were examined by SDS-PAGE and were shown in Fig. 3. The expression of the target protein was observed to be relatively high. Moreover, a clear and distinct protein band could be identified at 19.2 kDa, being the same as the theoretical molecular weight. The theoretical molecular weight of P-WT, P-m1, P-m2, P-C, P-N, P-Nm1, and P-Nm2 were 19.2 kDa, 19.2 kDa, 19.2 kDa, 20.4 kDa, 20.0 kDa, 20.0 kDa, 20.0 kDa, respectively.

### Optimal pH and the pH stability of mutants

**Fig. 4:**
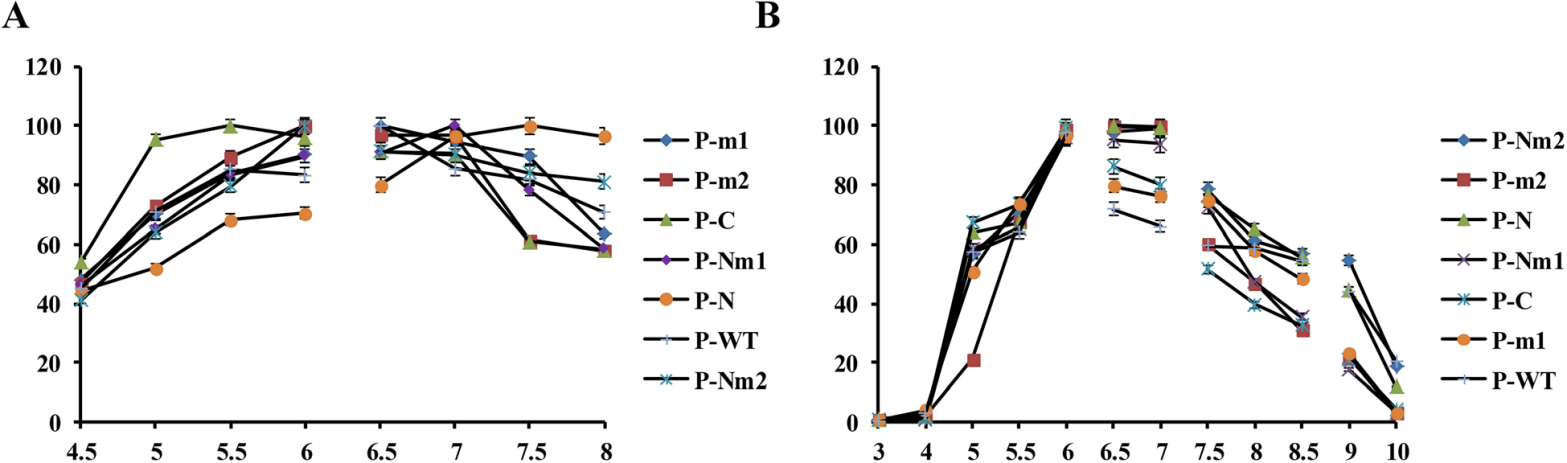
Effect of pH on P-WT and mutants. **A** Optimum pH; the best enzyme activity was defined to be 100%. **B** pH stability; the enzyme measured without incubation was defined to be 100%

The catalytic performance of the phenolic acid decarboxylase from various bacteria was reported to be very different [18], and the change of the pH value was previously observed to have effect on the growth of bacteria [19]. As shown in Fig. 4A, all enzymes exhibited optimal enzyme activity when the pH was close to neutral. P-C was found to be more active in acidic environments, whereas P-N was more active in alkaline environments. P-N and P-C showed optimal enzyme activity at pH 7.5 and 5.5, respectively. In addition, optimal enzyme activity for P-m1 was detected to be at pH 6.5, which was the same as for P-WT, whereas the optimal pH of P-Nm1 was found to be 7.0. Moreover, the optimal pH for the activity of P-m2 and P-Nm2 both showed to be at pH 6.0. As shown in Fig. 4B, the residual enzyme activity of P-N after incubation at pH 5.0 and 9.0 for 30 min were 64 and 44%, respectively. P-C was observed to have the strongest tolerance at pH 6.0, and the residual enzyme activity after incubation at pH 5.0 and pH 9.0 for 30 min were 67 and 21%, respectively. Furthermore, P-m1 had about 50% residual enzyme activity after being incubated in pH 5.0 and 8.5 for 30 min, respectively, as well as the residual enzyme activity was measured to be 20% after incubation in pH 9.0 for 30 min. 20% of the residual activity of P-m2 was detected to still remain after incubating in pH 5.0 and pH 9.0 for 30 min, respectively. Additionally, after incubation in pH 5.0 and pH 8.0 for 30 min, the residual enzyme activity of P-Nm1 was 58 and 40%, respectively. P-Nm2 was observed to have more than 50% enzyme activity after being incubated in pH 5.0 and pH 9.0 for 30 min, respectively, indicating that the N- terminus extension may be more conducive to the combination of the binding cavity with the substrate in an alkaline environment and may make its structure more stable. The phenolic acid decarboxylase isolated from *Lactobacillus brevis* RM84 was observed to have the best activity at pH 6.0 and was found to be stable in the range of pH 6.5 to 8.5 [20]. Furthermore, the optimal pH for the wild type as well as the mutants of FA decarboxylase from *Enterobacter* sp. Px6–4 was measured to be 7.0 [21]. The recombinant phenolic acid decarboxylase from *Cladosporium phlei* [22] and *B. pumilus* showed more than 90% enzyme activity between pH 5.0 and 6.0 [23].

### Optimum temperature and thermal stability

**Fig. 5:**
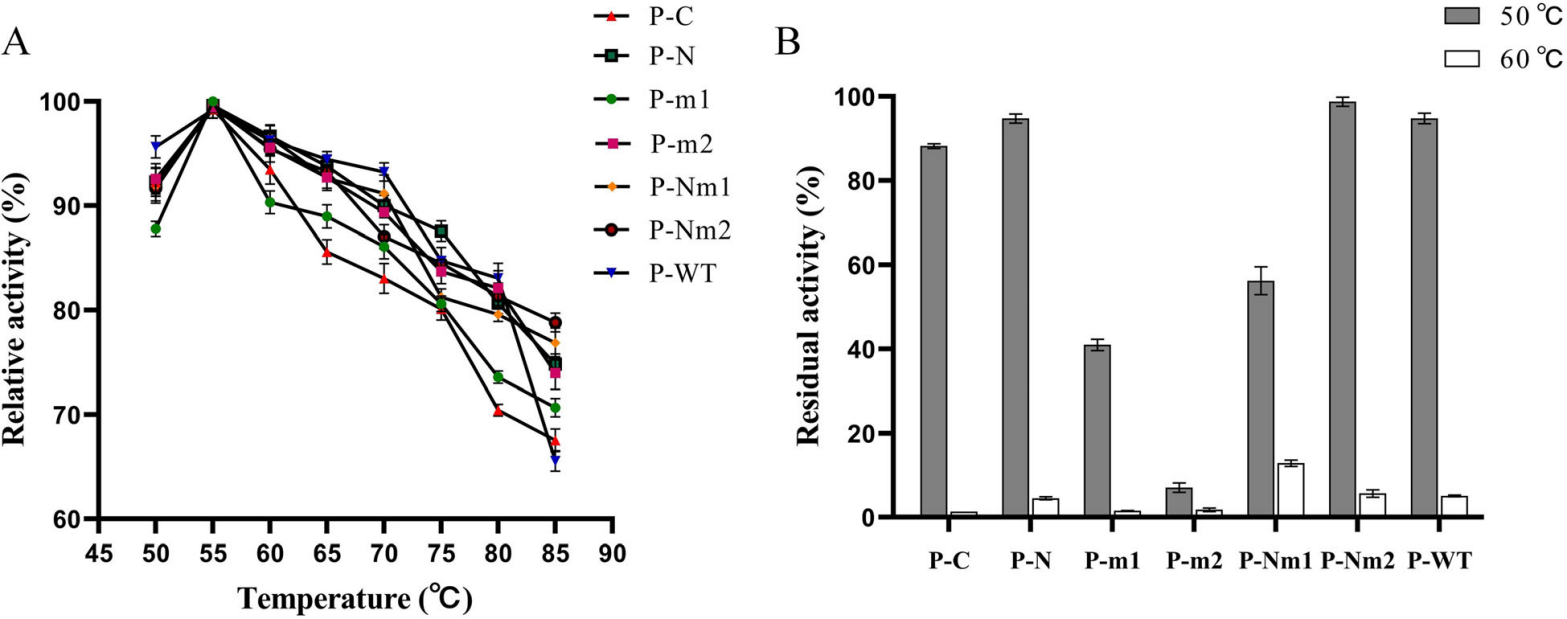
Effect of temperature on the P-WT and mutants. **A** Optimal temperature; the best enzyme activity was defined to be 100%. **B** Thermal stability; the enzyme measured without incubation was defined to be 100%

As shown in Fig. 5A, both mutants and P-WT showed the highest enzyme activity at 55 °C. As shown in Fig. 5B, after incubation at 50 °C and 60 °C for 60 min, the residual enzyme activity of P-N was 95 and 4.8%, respectively. In addition, the residual enzyme activity of P-C was detected to be 88 and 1.3% after being incubated at 50 °C and 60 °C for 60 min, respectively. Besides, the enzyme activity of P-m1 and P-m2 decreased after being kept at 50 °C for 60 min, among which P-m2 was almost inactivated. However, the residual enzyme activities of P-Nm1 and P-Nm2 were found to be greatly improved, as the residual enzyme activities after being incubated at 50 °C for 60 min were measured to be 58 and 99%, respectively. After being incubated at 60 °C for 60 min, the residual enzyme activities were still identified to be 13.4 and 6%, respectively, indicating that the extension of the N- terminus may increase the stability of the structure of the enzyme as well as increase its heat resistance. In comparison with *B. pumilus* (losing activity by incubation at 42 °C for 30 min), *Bacillus* sp. Bp-7 [24] (exhibiting 47% residual activity after incubating for 40 min at 50 °C), *L. brevis* (only 70% of the residual enzyme activity remaining after 1 h of storage at 37 °C) [20], *Cladosporium phlei* (decreasing to 75% after incubation for 5 min at 35 °C), *L. plantarum* (almost inactivating after incubation for 12 h at 30 °C) [25], *Candida guilliermondii* [22] (< 10% remaining after incubation at 50 °C for 20 min), P-WT and mutants were found to show good heat resistance. The enzyme that was stable and active in a wide range of pH and temperature might be advantageous in industrial applications [26].

### Specific activity and kinetic parameters

Both P-WT and mutants have been used to catalyze different substrates, PCA, FA, CA and SA, as shown in Table 1. Mutants were found to have a great change in the catalytic ability for four phenolic acids, and the value of specific activity was demonstrated to be the relative highest for the PCA and the lowest for the SA. This might be due to the obviously large parts in the two meta positions of the aromatic ring of the substrate (Fig. 1, the two methyl groups of the SA), which can affect the binding of the enzyme to the substrate [27].

**Table 1:** Specific activity of phenolic acid substrates of P-WT and mutants

	Specific activity (IU/mg)^a^			
	P-Coumaric acid	Ferulic acid	Caffeic acid	Sinapic acid
P-C	24.57 ± 0.18	3.41 ± 0.18	3.85 ± 0.38	1.26 ± 0.12
P-N	39.23 ± 0.26	7.84 ± 0.38	7.76 ± 0.08	2.69 ± 0.16
P-m1	42.97 ± 0.38	8.05 ± 0.31	9.32 ± 0.19	2.32 ± 0.08
P-m2	34.35 ± 0.28	4.43 ± 0.11	4.51 ± 0.33	1.49 ± 0.11
P-Nm1	57.49 ± 0.11	9.04 ± 0.26	10.54 ± 0.21	4.2 ± 0.19
P-Nm2	26.36 ± 0.19	5.22 ± 0.31	4.34 ± 0.18	0.36 ± 0.08
P-WT	13.44 ± 0.15	3.16 ± 0.32	2.87 ± 0.13	1.26 ± 0.06

^a^ These values were calculated by taking the average of three experiments conducted using SD

Table 2 summarized the kinetic parameters of six mutants and P-WT. Mutants were found to have a great change in the catalytic ability for four phenolic acids. The DOI number that submitted enzymology data to the STRENDA database was 10.22011/strenda_db.SYNCXF. The corresponding Michaelis-Menten curves were shown in the supplementary material. Comparison with P-WT, the *K*_m_ of P-C for the PCA, FA, and SA was reduced, especially for the PCA, the V_max_ of the three increased, as well as the *k*_cat_/*K*_m_ increased by 38, 71, 141%, which was consistent with the findings of previous reports stating the importance of the C-terminus region for substrate specificity or catalysis [8]. Although the truncated form of the C-terminus was observed to have the same length as a typical bacterial phenolic acid decarboxylase and had key active site residues, it also lacked function, which may be due to that the variable C-terminus region was important for substrate specificity or the catalytic mechanism [8, 14].

**Table 2:** Kinetic parameters of phenolic acid substrates of P-WT and mutants

		Kinetic constanta			
		P-Coumaric acid	Ferulic acid	Caffeic acid	Sinapic acid
P-C	V_max_ (IU/mg)	193.80 ± 2.38	97.73 ± 5.86	65.66 ± 4.79	12.36 ± 0.79
	*K*_m_ (mmol/L)	2.05 ± 0.29	1.03 ± 0.38	3.04 ± 0.69	1.31 ± 0.03
	*k*_cat_/*K*_m_ (mmol/L/s)	13.41 ± 0.45	28.15 ± 0.28	23.24 ± 0.52	40.87 ± 0.28
P-N	V_max_ (IU/mg)	683.10 ± 7.38	208.50 ± 8.38	143.80 ± 5.89	10.36 ± 0.34
	*K*_m_ (mmol/L)	7.02 ± 0.61	2.14 ± 0.83	1.48 ± 0.73	1.06 ± 0.42
	*k*_cat_/*K*_m_ (mmol/L/s)	14.53 ± 0.53	33.57 ± 0.49	37.57 ± 0.62	28.44 ± 0.53
P-m1	V_max_ (IU/mg)	17.26 ± 0.38	19.07 ± 8.39	124.60 ± 3.86	16.66 ± 0.78
	*K*_m_ (mmol/L)	1.88 ± 0.53	2.08 ± 0.28	1.36 ± 0.28	1.82 ± 0.38
	*K*_cat_/*K*_m_ (mmol/L/s)	152.38 ± 1.12	28.75 ± 0.39	32.14 ± 0.52	19.17 ± 0.19
P-m2	V_max_ (IU/mg)	136.20 ± 8.36	111.90 ± 10.91	72.64 ± 6.89	5.08 ± 0.58
	*K*_m_ (mmol/L)	1.22 ± 0.26	1.00 ± 0.38	6.49 ± 0.53	4.55 ± 0.61
	*k*_cat_/*K*_m_ (mmol/L/s)	15.97 ± 0.27	36.54 ± 0.36	4.07 ± 0.64	13.24 ± 0.66
P-Nm1	V_max_ (IU/mg)	254.40 ± 7.38	103.20 ± 7.48	87.37 ± 8.64	5.60 ± 0.53
	*K*_m_ (mmol/L)	3.52 ± 0.32	1.43 ± 0.06	1.21 ± 0.43	0.77 ± 0.64
	*k*_cat_/*K*_m_ (mmol/L/s)	11.31 ± 0.36	19.94 ± 0.73	24.72 ± 0.63	45.67 ± 0.25
P-Nm2	V_max_ (IU/mg)	186.30 ± 6.39	99.09 ± 0.3.83	144.20 ± 7.32	5.27 ± 0.17
	*K*_m_ (mmol/L)	12.05 ± 0.27	6.4 ± 0.38	9.33 ± 0.28	3.41 ± 0.26
	*k*_cat_/*K*_m_ (mmol/L/s)	10.33 ± 0.18	14.14 ± 0.59	10.38 ± 0.97	11.69 ± 0.11
P-WT	V_max_ (IU/mg)	121.50 ± 7.88	22.32 ± 4.31	89.46 ± 10.35	9.22 ± 0.19
	*K*_m_ (mmol/L)	4.09 ± 0.59	2.39 ± 0.58	3.10 ± 0.47	3.20 ± 0.30
	*k*_cat_/*K*_m_ (mmol/L/s)	9.74 ± 0.53	16.49 ± 0.42	9.76 ± 0.26	16.94 ± 0.58

^a^ These values were calculated by taking the average of three experiments conducted using SD

For P-N, the Vmax and the *k*_cat_/*K*_m_ for the FA, CA, and SA increased a lot, and the *K*_m_ value for the PCA increased from 4.09 mmol/L to 7.02 mmol/L. P-N showed better substrate affinities and higher turnover rates than P-WT for the FA, CA, and SA [28]. Also, previous research showed that the differences between different structures of the phenolic acid decarboxylase mainly exist in the random coils at the N-terminus as well as the C-terminus of the protein [11], which may play a key role in the catalytic process of these decarboxylases [13]. In Gao’s experiment, the N-terminus truncation of the phenolic acid decarboxylase resulted in a lack of activity against any substrate, which may be due to the lack of the four conserved catalytic residues [8].

P-m1 mutation from the His92 to Glu92 resulted in a reduction of the *k*_m_ for four phenolic acids from 4.09 mmol/L, 2.39 mmol/L, 3.10 mmol/L, 3.20 mmol/L to 1.88 mmol/L, 2.08 mmol/L, 1.36 mmol/L, 1.82 mmol/L, as well as for the mutation from the Tyr122 to Leu122 resulted in the alteration of the *K*_m_ for four substrates to 1.22 mmol/L, 1.00 mmol/L, 6.49 mmol/L, 4.55 mmol/L, whereas the *k*_cat_/*K*_m_ of these four increased by 56, 74, 229, 113% respectively. The docking data of a previous study suggested that the changes involving the residues with different charges may affect the orientation of the matrix in the cavity before entering the active site [17]. In comparison with P-WT, the affinity of P-m1 to four substrates as well as P-m2 to the PCA and the FA showed a great improvement, however, the affinity of P-m2 to the CA and SA decreased. The presence of specific negatively charged residues in the substrate-binding cavity was found to increase the affinity of the enzyme to the substrate, which behavior was also demonstrated to be present in other enzymes, for example in association with the interaction of ginkgo salicylic acid and the salicylic acid decarboxylase [29]. Besides, it was reported that there is a common cavity or gap between the two pieces of each subunit [16] and that the cavity becomes larger near the molecular surface and gradually narrows as the enzyme enters. The entry region was reported to be comprised of the amino acid side chains Asn22 (15) (original P-WT numbering in bracket), Trp24 (17), Met44 (37), Glu99 (92), Pro102 (95), Leu129 (122), Val131 (124), and Glu133 (126) [9]. Sequence comparison of a large number of active sites identified that a certain degree of variation was shown by some sites in the substrate-binding cavity, especially E99 (92), L129 (122), and V131 (124), which were closely related to the combination of the enzyme and the substrate and the catalytic mechanism [17].

In this study, the mutation H92E (99) formed E-Y-V with the 122Y and the 124 V, as well as the mutation Y122L formed H-L-V with the 92H and the 124 V. From the V_max_ and *K*_m_ values it can be concluded that they have a stronger affinity for the substrate in comparison with the original H-Y-V of P-WT, which finding was consistent with that of a previous report [8]. The structure of the E-Y-V and the H-L-V could both form a hydrogen bond with the substrate. The original H-Y-V structure caused the hydrogen bond with the substrate to disappear, which was very unstable at this time. This finding was consistent with a previously conducted detailed analysis on the molecular interactions between the substrate-binding cavity and the trans-coumaric acid in these structures, which may be caused by the steric effect of a large number of the residues H94 (101) and Y124 (131) [17].

The *K*_m_ value of P-Nm1 in comparison with those of the four phenolic acids was found to be all decreased. Perhaps the extension of the N-terminal contributed to the increase of the substrate-binding pocket, allowing it to accept more substrates [11]. However, the *K*_m_ of P-Nm2 increased to 12.05 mmol/L, 6.4 mmol/L, 9.33 mmol/L, and 3.41 mmol/L, indicating that P-Nm2 reduced the affinity to the four phenolic acids, which was quite different finding from what was expected. However, it can be seen from Tables 1 and 2 that the phenolic acid decarboxylase used in this study also had great decarboxylation ability for SA in comparison with other enzymes. Sinapic acid can be described with possessing a methoxy group at position 5 along with its decarboxylation product being canolol (4-vinylsyringol) [18], which has physiological functions, including anti-oxidation, anti-cancer, and anti-recombination [30] in addition to having high nutritional and medical value. The phenolic acid decarboxylase with this characters might be developed into a potential biocatalyst for the production of rapeseed polyphenols, which could have certain application value [31]. To the best of our knowledge, improving catalytic characteristics of phenolic acid decarboxylase by engineering N- and C-terminal region have never been reported.

## Conclusion

In this study, C-terminal extensions, N-terminal extensions, and the combination of N-terminal extensions and H92E as well as Y122L were successfully synthesized. Compared with P-WT, P-C was more active in the acidic environment, whereas the extension of the N-terminus contributed to the alkali resistance and heat resistance. The catalytic performance of these derivatives is considered to be an improvement because its alkali resistance and heat resistance make these enzymes excellent candidates for further applications. And it is necessary to make further efforts to improve the catalytic characters by clarifying the relationship between the amino acid sequence of these enzymes and the molecular structure.

## Materials and methods

### Gene, stains, vectors, and substrates

*Bacillus amyloliquefaciens* ZJH-01 was preserved in our laboratory. The Bacterial DNA Extraction Kit and Gel Extraction Kit were purchased from Omega. Taq polymerase as well as plasmid pMD18-T were purchased from Takara. T4 DNA ligase and Q5® High-Fidelity DNA polymerase were purchased from Biolabs. Plasmid Miniprep Kit was purchased from Biomiga.

### Cloning and overexpression

The total genome was extracted from *Bacillus amyloliquefaciens* ZJH-01 using a Bacterial DNA Extraction Kit, and primers were designed based on phenolic acid decarboxylase gene sequences retrieved from NCBI (Accession code: 014305882) to obtain the target gene, as shown in Table S1 in the Supplementary data. The target fragment obtained by the PCR was ligated with the cloning vector pMD18-T and subsequently transfected into *E. coli* DH-5α. 100 μL 100 mg/mL ampicillin antibiotics was added to 100 mL medium. The gene with the correct sequencing was ligated with pET-28a (+) at the *Nco*I and *Xho*I restriction sites and then transferred to *E. coli* BL21 (DE3). Eventually, 100 μL 100 mg/mL kana antibiotic was added to 100 mL medium.

### Production and purification

The recombinant gene was transferred to LB medium and cultured at 200 rpm, 37 °C, and subsequently induced with 200 μL 500 mmol/L Isopropyl β-D-1-thiogalactopyranoside (IPTG) for expression. The bacterial solution was afterwards centrifuged at 5000 rpm, 4 °C for 5 min, and 10 mL 50 mmol/L Tris-HCl buffer (pH 7.0) was added after discarding the supernatant. The crude enzyme solution was obtained by ultrasonication for 15 min and centrifugation was used to separate the supernatant at 10,000 rpm for 10 min. The crude enzyme solution was purified on the ÄKTA FPLC purification system using a nickel sepharose column (1 × 10 cm) and 50 mmol/L phosphate buffer (pH 7.8) consisting of 300 mmol/L NaCl and different concentrations (40, 45, 250 mmol/L) of imidazole [32]. The molecular weight and concentration of the protein were determined by sodium dodecyl sulfate-polyacrylamide gel electrophoresis (SDS-PAGE) and a bicinchoninic acid kit (BCA Kit), respectively.

### Assay of enzyme activity

Regarding the definition of enzyme activity, 1 IU refers to the amount of enzyme that produces 1 μmol of 4-vinylbenzene per minute. As for the assay of enzyme activity, 0.8 mL of 200 mmol/L Na2HPO4-citrate buffer (pH 6.0), 0.1 mL of 50 mmol/L P-Coumaric acid, and 0.1 mL of phenolic acid decarboxylase were added to the reaction system. After the reaction took place at 37 °C for 5 min, 2 ml of methanol was added to terminate it. The 4-vinylphenol content formed was determined by high-performance liquid chromatography (HPLC) after the filtration of the content through a 0.22 μmol filter. The 0.1 mL enzyme solution was replaced with 0.1 mL buffer as a blank control. The HPLC model was an UltiMate 3000 high-performance liquid chromatography using a Hypersil GOLD C18 Selective LC column with a packing diameter of 3 μm and a size of 4.6 mm × 250 mm. The flow rate was set to 1.0 mL/min and the column temperature was selected to be 30 °C. The mobile phase of the PCA was methanol: 0.1% acetic acid = 50: 50, and the detection wavelength was adjusted to be 280 nm. The mobile phase of the FA was methanol: 0.1% acetic acid = 40: 60, and the detection wavelength was 310 nm. The mobile phase of the CA was methanol: 0.1% acetic acid = 40: 60, and the detection wavelength was 280 nm. The mobile phase of the SA was methanol: 0.1% acetic acid = 50: 50, and the detection wavelength was 310 nm.

### Optimum pH and pH stability

The purified enzyme was reacted under different pH (200 mmol/L citrate buffer for pH 4.5–6.0 and 200 mmol/L Na_2_HPO_4_-NaH_2_PO_4_ for pH 6.5–8.0) to determine its optimal pH, using p-coumaric acid as a substrate. The best enzyme activity was defined to be 100%. The purified enzyme was incubated under different pH (200 mmol/L citrate buffer for pH 3.0–6.0 and 200 mmol/L Na_2_HPO_4_-NaH_2_PO_4_ for pH 6.5–7.0, 200 mmol/L Tris-HCl for pH 7.5–8.5, 200 mmol/L Gly-NaOH for pH 9.0–10.0) at 37 °C for 30 min, and the residual enzyme activity was measured in the optimal pH to study the pH stability of the enzyme. The enzyme activity of the untreated enzyme was observed to be 100%. All experiments were done three times.

### Optimum temperature and thermal stability

The purified enzymes were reacted at various temperatures (50 °C–85 °C) for the determination of the optimal temperature, using p-coumaric acid as a substrate. The best enzyme activity was defined to be 100%. After incubating for 60 min at different temperatures (50 °C, 60 °C), the residual enzyme activity was measured at 37 °C with the standard method for determination enzyme activity. The enzyme activity of the untreated enzyme was 100%. All experiments were done three times.

### Specific activity and kinetic parameters

The specific unit of enzyme activity was defined as the unit of enzyme activity (IU/mg) per mg of enzyme protein. The amount of protein added was determined by the BCA method, subsequently calculating the specific enzyme activity for the different substrates. 5 mmol/L PCA, 5 mmol/L FA, 5 mmol/L CA, as well as 5 mmol/L SA were respectively used as the substrate to react at 37 °C for 5 min in order to study the associated substrate specificity. The kinetic parameters were determined by the addition different concentrations of the substrates. The V_max_ and the *K*_m_ of the enzyme were calculated by nonlinear fitting using the GraphPad Prism 5.0 software and the *k*_cat_ value was calculated according to the theoretical molecular weight of the phenolic acid decarboxylase enzyme. All experiments were done three times.

## Supplementary Information

**Additional file 1.**

## Data Availability

All data generated or analyzed during this study were included in this published article and its supplementary information files.
