# Supplementary material for: Improving the catalytic characteristics of phenolic acid decarboxylase from Bacillus amyloliquefaciens by the engineering of N-terminus and C-terminus
Source: BMC Biotechnol. 2021 Jul 26;21:44. doi: 10.1186/s12896-021-00705-7 (PMC8311932; doi:10.1186/s12896-021-00705-7)
Supplement: Supplementary file 1 — Additional file 1. [file 12896_2021_705_MOESM1_ESM.docx]

**Improving the catalytic characteristics of phenolic acid decarboxylase from *Bacillus amyloliquefaciens* by the engineering of N-terminus and C-terminus**

**Qin Li^123^, Ying Xia^12^, Ting Zhao^2^, Yuanyuan Gong^2^, Shangling Fang^2*^, Maobin Chen^2*^**

1. These authors contribute equal to this work.
2. Key Laboratory of Fermentation Engineering(Ministry of Education), Hubei Provincial Cooperative Innovation Center of Industrial Fermentation, Hubei Key Laboratory of Industrial Microbiology, School of Food and Biological Engineering, Hubei University of Technology, No.28, Nanli Road, Wuhan, 430068, China
3. Sichuan Agricultural University, No.46, Xinkang Road, Yaan, 625014, China.

^*^Corresponding author:

Maobin Chen, Professor

School of Food and Biological Engineering,Hubei University of Technology, No.28, Nanli Road, Wuhan, 430068, China. Phone: +86-027-59750483; Fax number: +86-027-59750009; E-mail: [hgchenmaobin@163.com](mailto:hgchenmaobin@163.com)

**Table S1**

| Primers | Sequences (5’ - 3’) |
| --- | --- |
| P-NcoI | 5′-CATGCCATGGCCATGGAAAACTTTATCGGAAGCCATA-3′ |
| P-XhoI | 5′-CCGCTCGAGTTTTAATTTTCCCGCGCGAATA-3′ |
| NFuf | 5’-CATGCCATGGCC**ATGACAAAAACTTTTAAAACACTTGATGACTTTCTC**GGAAGCCATATGATTTACAC-3’ |
| N-F | 5’-CATGCCATGGCCATGACAAAAACTTTTAAAACACTTGATGA-3’ |
| CFur | 5’-CCGCTCGAG**CTTATTTAGACGATGGTAGTTTTGATCAAAGTACTTGCCGTT**GCGAATATCATCGGT-3’ |
| C-R | 5’-CCGCTCGAGCTTATTTAGACGATGGTAGTTTTGATCA-3’ |
| M1f | 5’-CCCGAAATGGGTGGAAGAGCATCCGG-3’ |
| M1r | 5’-CCGGATGCTCTTCCACCCATTTCGGG-3’ |
| M2f | 5’-CGTACCCGAAACTAGTCGTTCCCGAG-3’ |
| M2r | 5’-CTCGGGAACGACTAGTTTCGGGTACG-3’ |

The primers P-NcoI and P-XhoI are used to clone the full-length of phenolic acid decarboxylase from *Bacillus amyloliquefaciens* (Accession code: 014305882). Primers NFuf and N-F are used to replace the N-terminal terminal of phenolic acid decarboxylase. Primers CFur and C-R are used to replace the C-terminal terminal of phenolic acid decarboxylase. The substituted N-terminal and C-terminal are shown in bold. M1f, M1r, M2f and M2r are used to introduce site-directed mutagenesis, the mutated sites are shown in red.

**Table S2**

| Primers | Sequences (5’ - 3’) |
| --- | --- |
| P-C | MENFIGSHMI YTYENGWEYE IYIKNDHTID YRIHSGMVGG RWVRDQEVNI VKLTEGVYKV SWTEPTGTDV SLNFMPNEKR MHGIIFFPKW VHEHPEITVC YQNDYIDVMK ESREKYETYP KYVVPEFADI TYLNNAGINN ETLISEAPYE GMTDDIRNGK YFDQNYHRLN K |
| P-N | MTKTFKTLDD FLGSHMIYTY ENGWEYEIYI KNDHTIDYRI HSGMVGGRWV RDQEVNIVKL TEGVYKVSWT EPTGTDVSLN FMPNEKRMHG IIFFPKWVHE HPEITVCYQN DYIDVMKESR EKYETYPKYV VPEFADITYL NNAGINNETL ISEAPYEGMT DDIRAGKLK |
| P-m1 | MENFIGSHMI YTYENGWEYE IYIKNDHTID YRIHSGMVGG RWVRDQEVNI VKLTEGVYKV SWTEPTGTDV SLNFMPNEKR MHGIIFFPKW VEEHPEITVC YQNDYIDVMK ESREKYETYP KYVVPEFADI TYLNNAGINN ETLISEAPYE GMTDDIRAGK LK |
| P-m2 | MENFIGSHMI YTYENGWEYE IYIKNDHTID YRIHSGMVGG RWVRDQEVNI VKLTEGVYKV SWTEPTGTDV SLNFMPNEKR MHGIIFFPKW VHEHPEITVC YQNDYIDVMK ESREKYETYP KLVVPEFADI TYLNNAGINN ETLISEAPYE GMTDDIRAGK LK |
| P-Nm1 | MTKTFKTLDD FLGSHMIYTY ENGWEYEIYI KNDHTIDYRI HSGMVGGRWV RDQEVNIVKL TEGVYKVSWT EPTGTDVSLN FMPNEKRMHG IIFFPKWVEE HPEITVCYQN DYIDVMKESR EKYETYPKYV VPEFADITYL NNAGINNETL ISEAPYEGMT DDIRAGKLK |
| P-Nm2 | MTKTFKTLDD FLGSHMIYTY ENGWEYEIYI KNDHTIDYRI HSGMVGGRWV RDQEVNIVKL TEGVYKVSWT EPTGTDVSLN FMPNEKRMHG IIFFPKWVHE HPEITVCYQN DYIDVMKESR EKYETYPKLV VPEFADITYL NNAGINNETL ISEAPYEGMT DDIRAGKLK |
| P-WT | MENFIGSHMI YTYENGWEYE IYIKNDHTID YRIHSGMVGG RWVRDQEVNI VKLTEGVYKV SWTEPTGTDV SLNFMPNEKR MHGIIFFPKW VHEHPEITVC YQNDYIDVMK ESREKYETYP KYVVPEFADI TYLNNAGINN ETLISEAPYE GMTDDIRAGK LK |


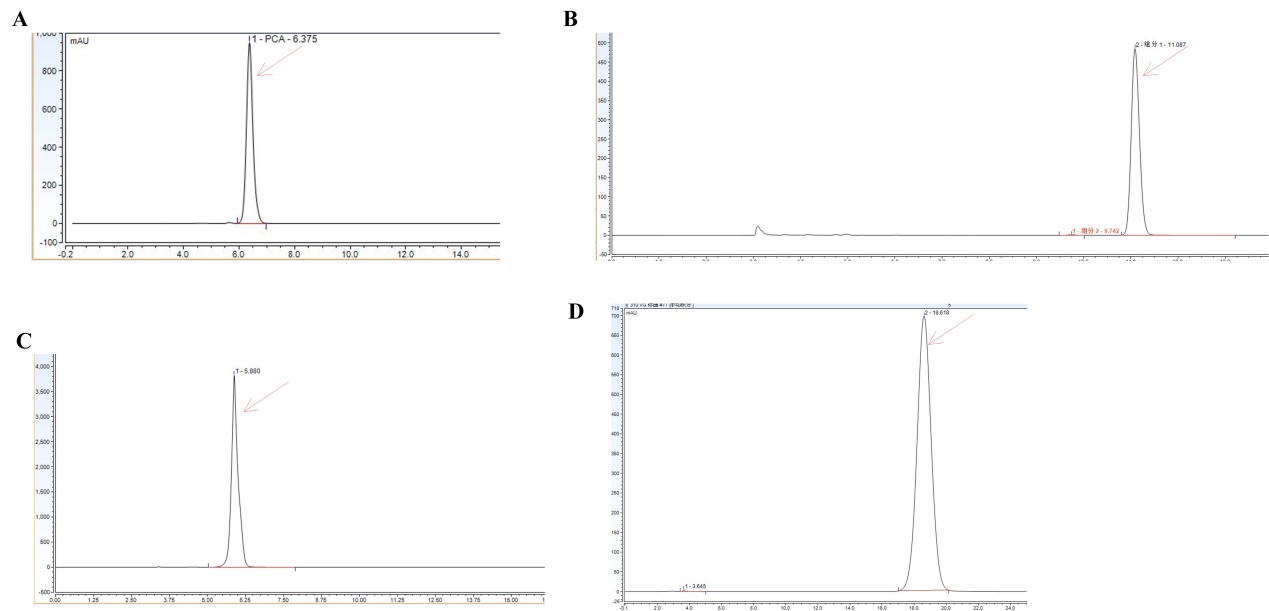


**Fig.S1**. A: (A) p-coumaric acid. (B) 4-vinyl phenol. (C) Ferulic acid. (D) Vinyl guaiacol


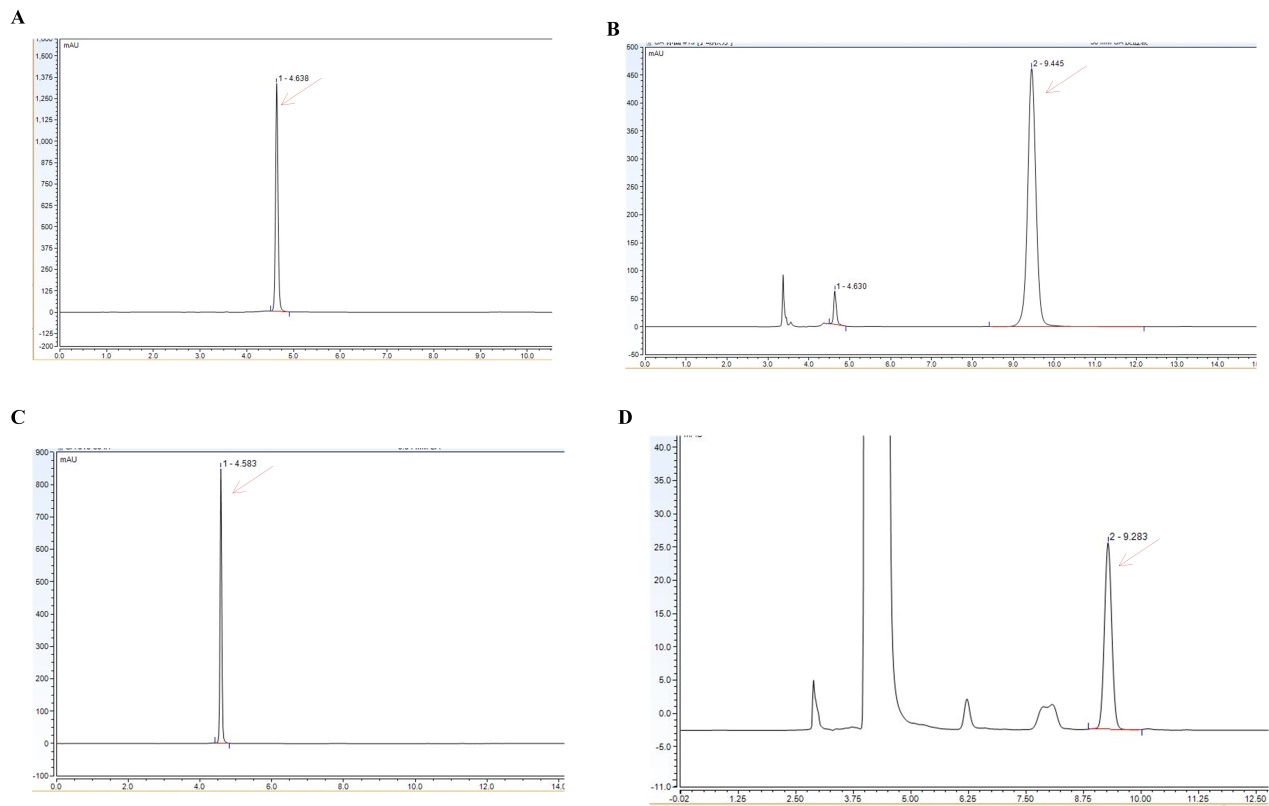


**Fig.S2** (A) Caffeic acid. (B) 4-vinyl benzene (C) Sinapic acid. (D) 4-vinylsyringol


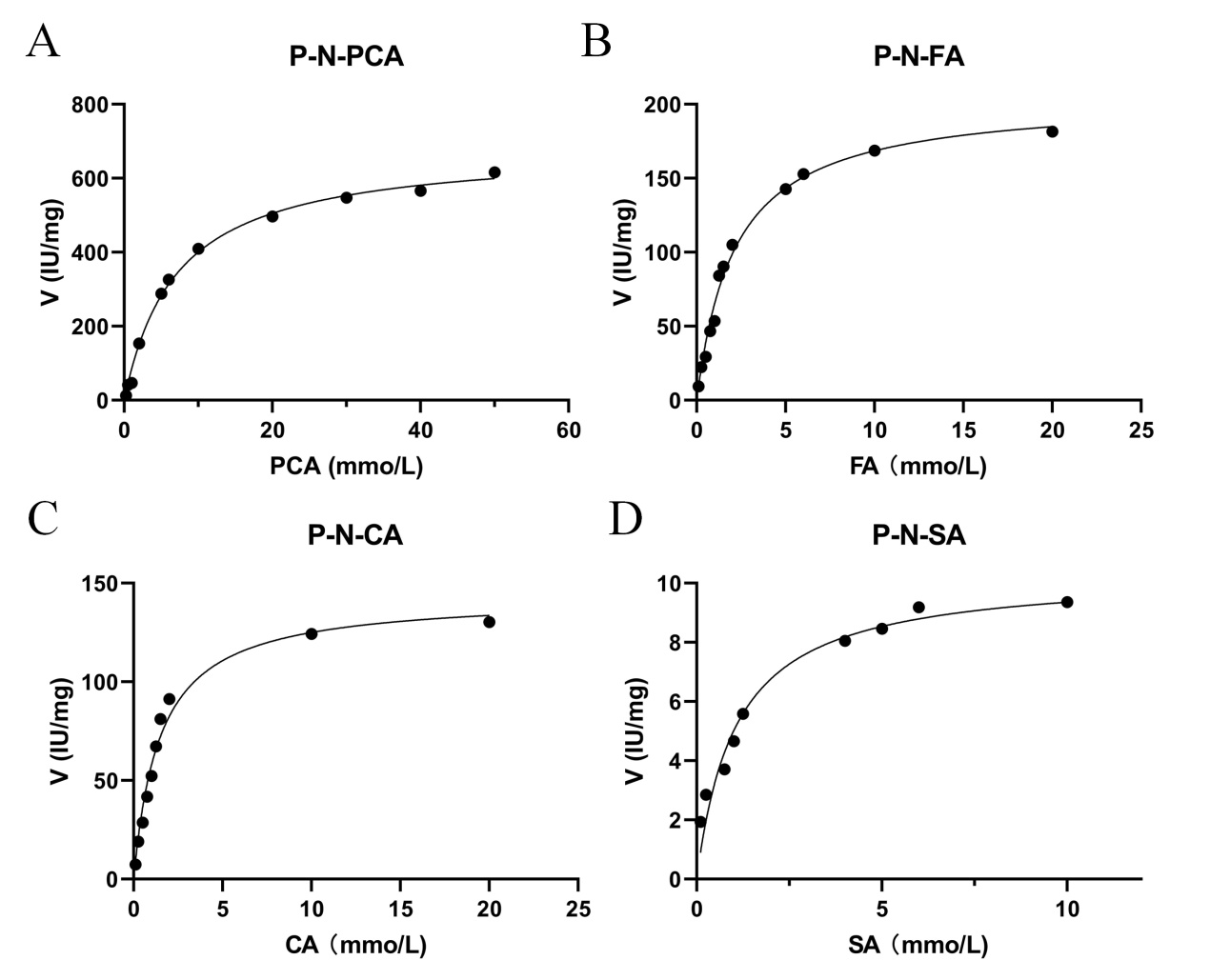


**Fig.S3** The Michaelis-Menten of P-N (A) p-coumaric acid. (B) Ferulic acid. (C) Caffeic acid. (D) Sinapic acid

**Table S3** Kinetic parameters of P-N

|  | P-Coumaric acid | Ferulic acid | Caffeic acid | Sinapic acid |
| --- | --- | --- | --- | --- |
| V_max_(IU/mg) | 683.10±7.38 | 208.50±8.38 | 143.80±5.89 | 10.36±0.34 |
| *K*_m_(mmol/L) | 7.02±0.61 | 2.14±0.83 | 1.48±0.73 | 1.06±0.42 |
| *k*_cat_(S^-1^) | 102±3.75 | 71.84±3.11 | 55.60±2.12 | 45.50±0.92 |
| *k*_cat_/*K*_m_(mmol/L/s) | 14.53±0.53 | 33.57±0.49 | 37.57±0.62 | 28.44±0.53 |

The protein concentration of P-N was 0.9734 mg/mL.


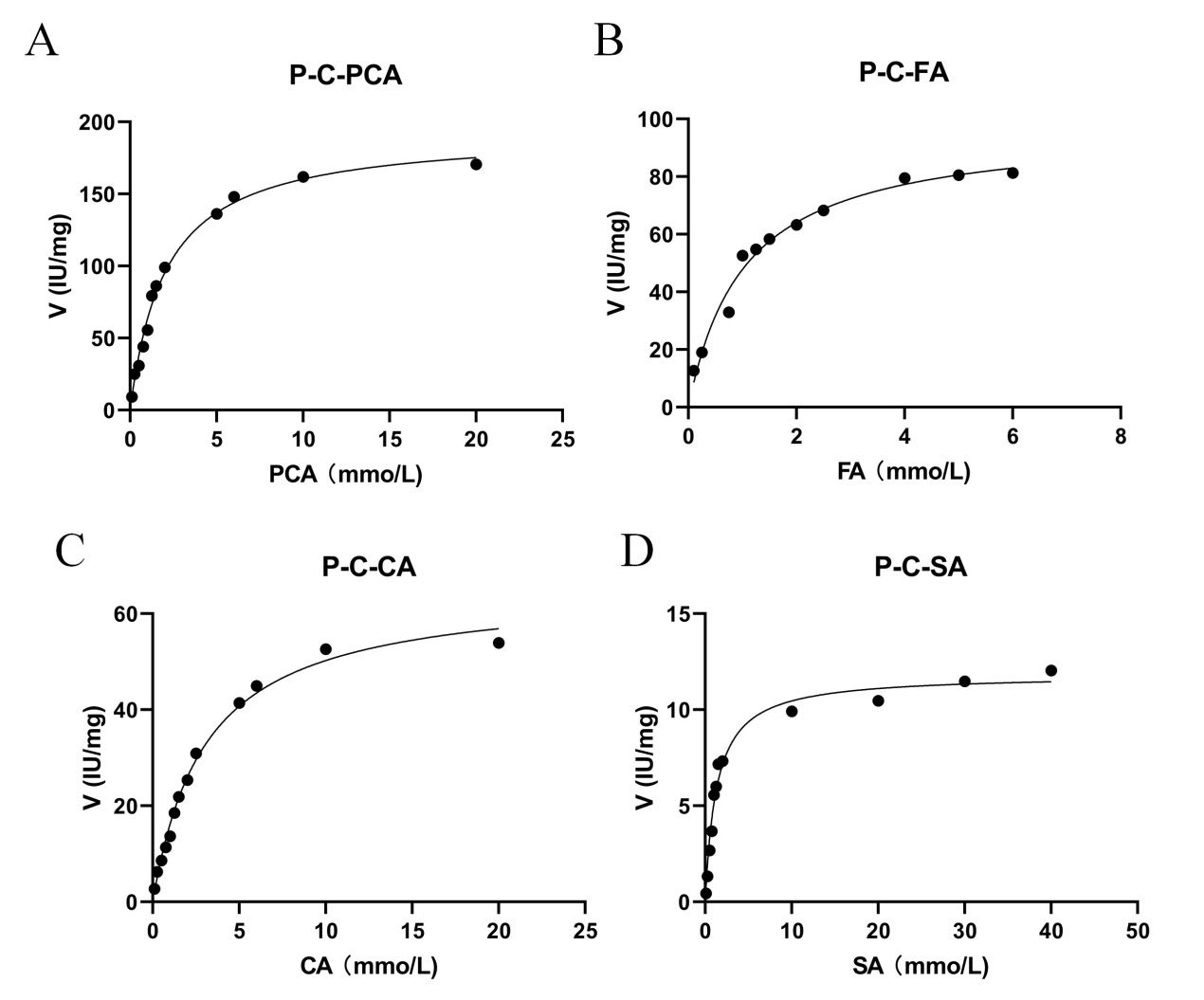


**Fig.S4** The Michaelis-Menten of P-C (A) p-coumaric acid. (B) Ferulic acid. (C) Caffeic acid. (D) Sinapic acid

**Table S4** Kinetic parameters of P-C

|  | P-Coumaric acid | Ferulic acid | Caffeic acid | Sinapic acid |
| --- | --- | --- | --- | --- |
| V_max_(IU/mg) | 193.80±2.38 | 97.73±5.86 | 65.66±4.79 | 12.36±0.79 |
| *K*_m_(mmol/L) | 2.05±0.29 | 1.03±0.38 | 3.04±0.69 | 1.31±0.03 |
| *k*_cat_(S^-1^) | 27.49±0.26 | 28.99±0.83 | 70.65±2.21 | 53.54±1.28 |
| *k*_cat_/*K*_m_(mmol/L/s) | 13.41±0.45 | 28.15±0.28 | 23.24±0.52 | 40.87±0.28 |

The protein concentration of P-C was 1.5058 mg/mL.


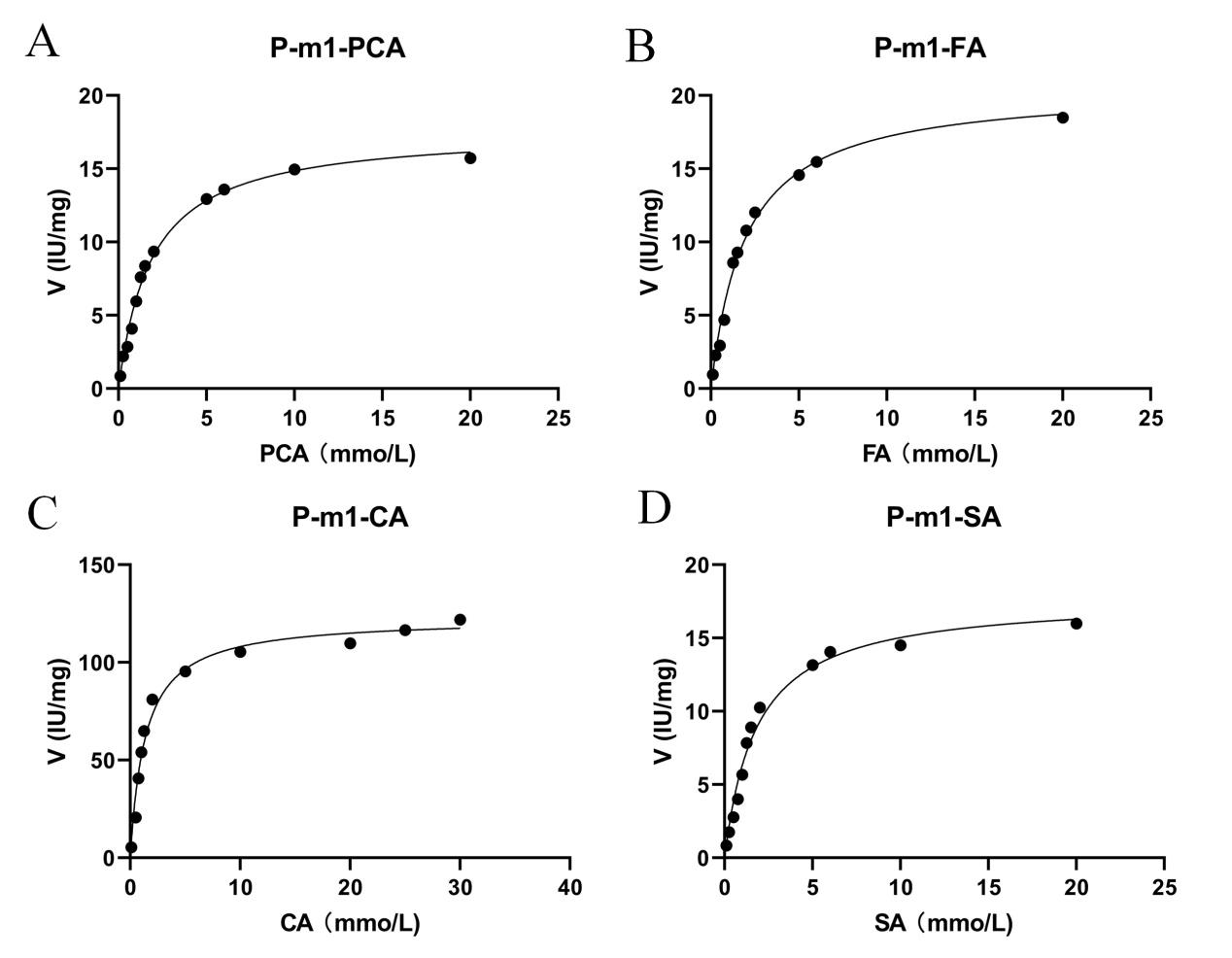


**Fig.S5** The Michaelis-Menten of P-m1 (A) p-coumaric acid. (B) Ferulic acid. (C) Caffeic acid. (D) Sinapic acid

**Table S5** Kinetic parameters of P-m1

|  | P-Coumaric acid | Ferulic acid | Caffeic acid | Sinapic acid |
| --- | --- | --- | --- | --- |
| V_max_(IU/mg) | 17.26±0.38 | 19.07±8.39 | 124.60±3.86 | 16.66±0.78 |
| *K*_m_(mmol/L) | 1.88±0.53 | 2.08±0.28 | 1.36±0.28 | 1.82±0.38 |
| *k*_cat_(S^-1^) | 286.47±5.78 | 59.80±1.71 | 43.71±0.82 | 34.89±0.38 |
| *k*_cat_/*K*_m_(mmol/L/s) | 152.38±1.12 | 28.75±0.39 | 32.14±0.52 | 19.17±0.19 |

The protein concentration of P-m1 was 0.9196 mg/mL.


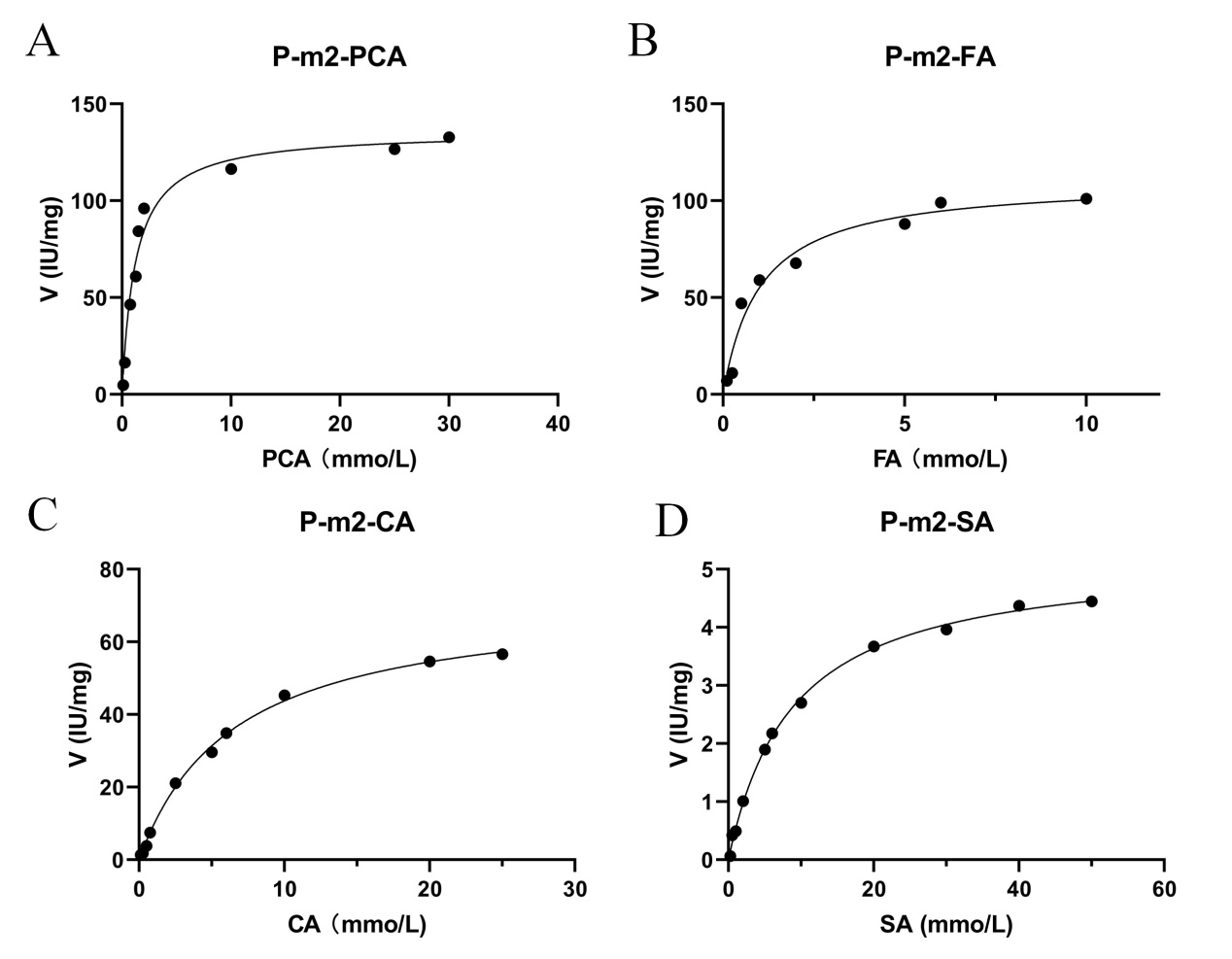


**Fig.S6** The Michaelis-Menten of P-m2 (A) p-coumaric acid. (B) Ferulic acid. (C) Caffeic acid. (D) Sinapic acid

**Table S6** Kinetic parameters of P-m2

|  | P-Coumaric acid | Ferulic acid | Caffeic acid | Sinapic acid |
| --- | --- | --- | --- | --- |
| V_max_(IU/mg) | 136.20±8.36 | 111.90±10.91 | 72.64±6.89 | 5.08±0.58 |
| *K*_m_(mmol/L) | 1.22±0.26 | 1.00±0.38 | 6.49±0.53 | 4.55±0.61 |
| *k*_cat_(S^-1^) | 19.48±0.26 | 36.54±0.41 | 26.41±0.28 | 60.24±1.38 |
| *k*_cat_/*K*_m_(mmol/L/s) | 15.97±0.27 | 36.54±0.36 | 4.07±0.64 | 13.24±0.66 |

The protein concentration of P-m2 was 1.1185 mg/mL.


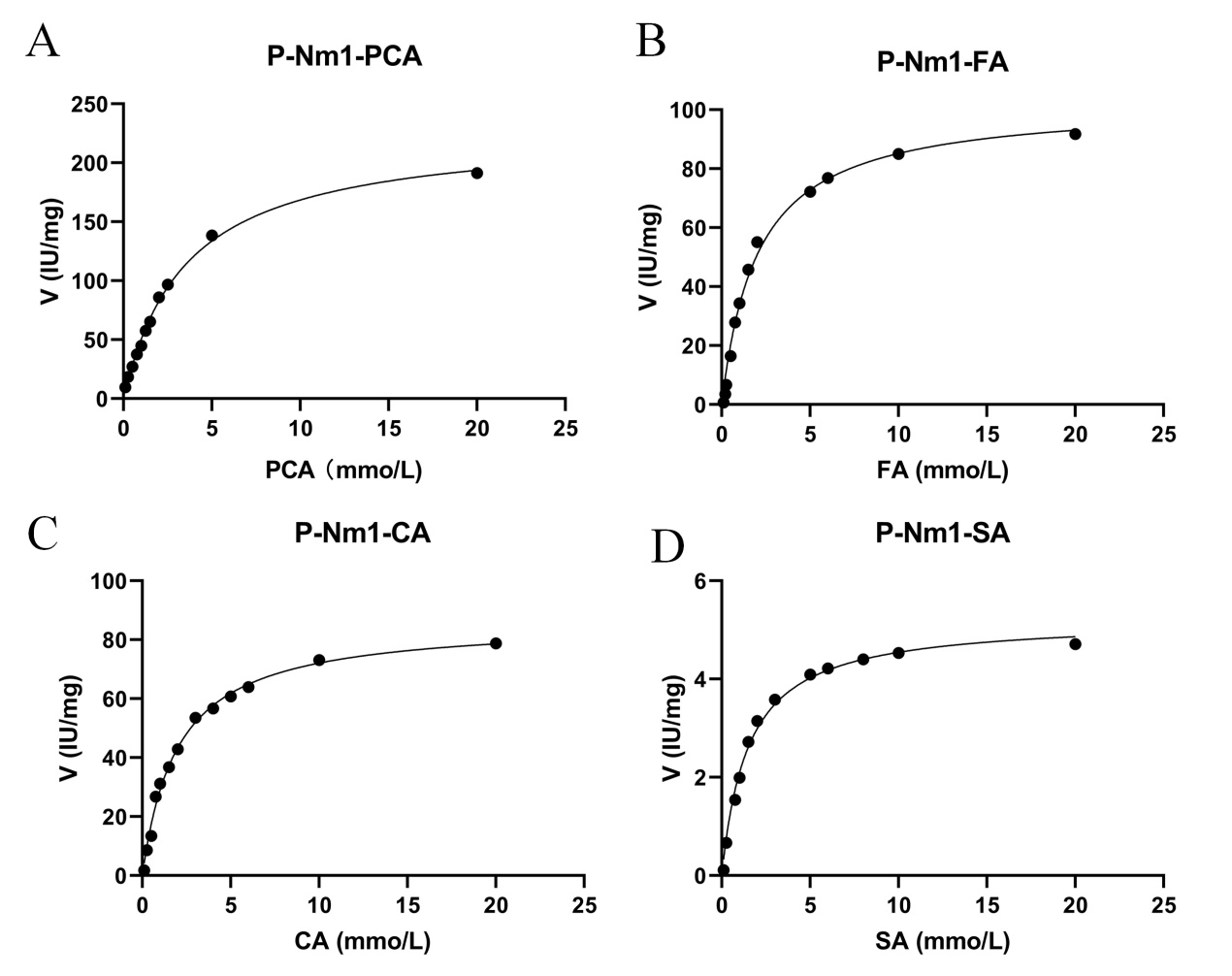


**Fig.S7** The Michaelis-Menten of P-Nm1 (A) p-coumaric acid. (B) Ferulic acid. (C) Caffeic acid. (D) Sinapic acid

**Table S7** Kinetic parameters of P-Nm1

|  | P-Coumaric acid | Ferulic acid | Caffeic acid | Sinapic acid |
| --- | --- | --- | --- | --- |
| V_max_(IU/mg) | 254.40±7.38 | 103.20±7.48 | 87.37±8.64 | 5.60±0.53 |
| *K*_m_(mmol/L) | 3.52±0.32 | 1.43±0.06 | 1.21±0.43 | 0.77±0.64 |
| *k*_cat_(S^-1^) | 39.81±0.52 | 28.51±0.28 | 29.91±0.29 | 35.17±0.72 |
| *k*_cat_/*K*_m_(mmol/L/s) | 11.31±0.36 | 19.94±0.73 | 24.72±0.63 | 45.67±0.25 |

The protein concentration of P-Nm1 was 0.7234 mg/mL.


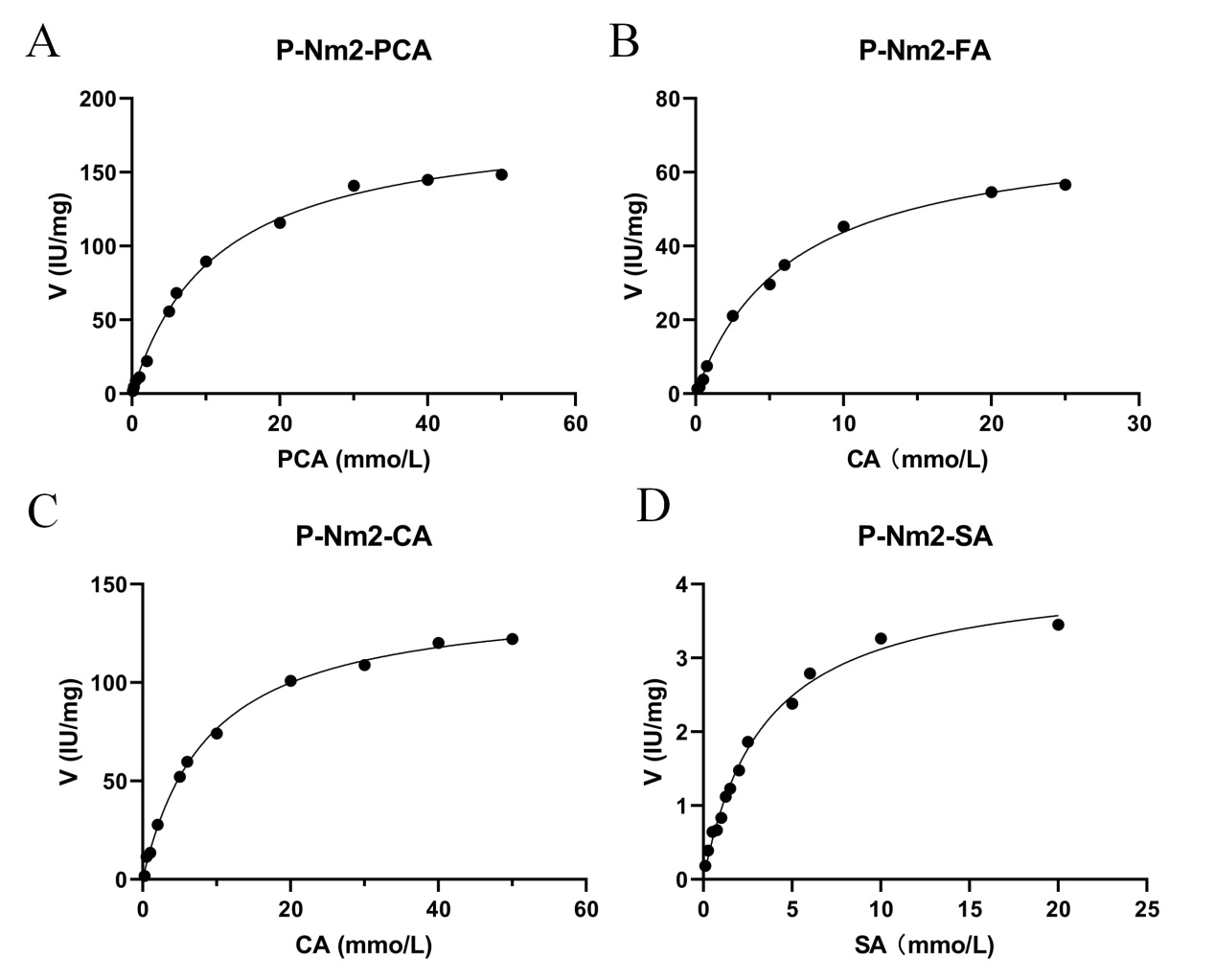


**Fig.S8** The Michaelis-Menten of P-Nm2 (A) p-coumaric acid. (B) Ferulic acid. (C) Caffeic acid. (D) Sinapic acid

**Table S8** Kinetic parameters of P-Nm2

|  | P-Coumaric acid | Ferulic acid | Caffeic acid | Sinapic acid |
| --- | --- | --- | --- | --- |
| V_max_(IU/mg) | 186.30±6.39 | 99.09±0.3.83 | 144.20±7.32 | 5.27±0.17 |
| *K*_m_(mmol/L) | 12.05±0.27 | 6.4±0.38 | 9.33±0.28 | 3.41±0.26 |
| *k*_cat_(S^-1^) | 124.48±2.49 | 90.50±5.21 | 96.85±4.29 | 39.86±3.01 |
| *k*_cat_/*K*_m_(mmol/L/s) | 10.33±0.18 | 14.14±0.59 | 10.38±0.97 | 11.69±0.11 |

The protein concentration of P-Nm2 was 1.5460 mg/mL.


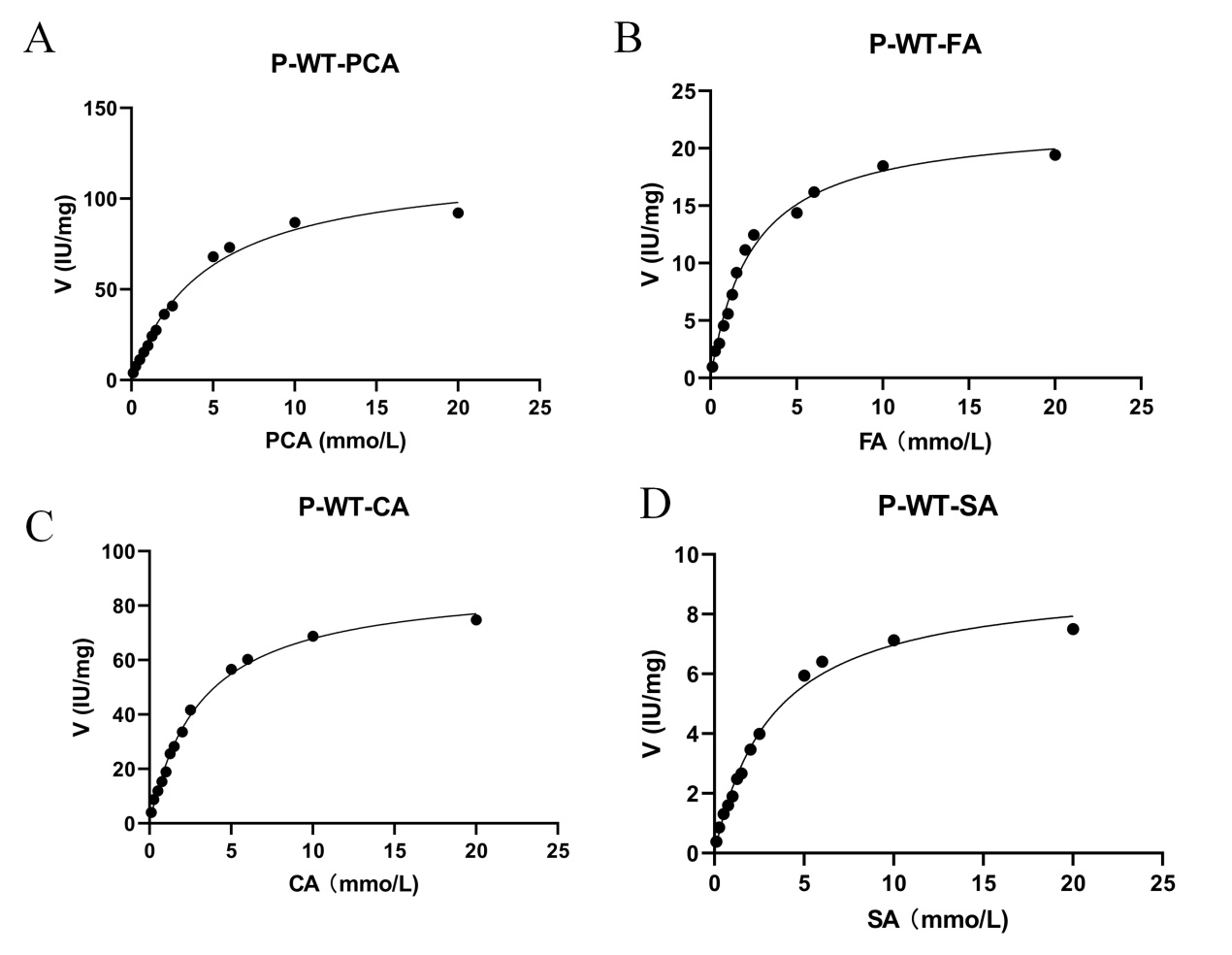


**Fig.S9** The Michaelis-Menten of P-WT (A) p-coumaric acid. (B) Ferulic acid. (C) Caffeic acid. (D) Sinapic acid

**Table S9** Kinetic parameters of P-WT

|  | P-Coumaric acid | Ferulic acid | Caffeic acid | Sinapic acid |
| --- | --- | --- | --- | --- |
| V_max_(IU/mg) | 121.50±7.88 | 22.32±4.31 | 89.46±10.35 | 9.22±0.19 |
| *K*_m_(mmol/L) | 4.09±0.59 | 2.39±0.58 | 3.10±0.47 | 3.20±0.30 |
| *k*_cat_(S^-1^) | 39.84±0.58 | 39.41 | 30.26±0.72 | 54.21±2.18 |
| *k*_cat_/*K*_m_(mmol/L/s) | 9.74±0.53 | 16.49±0.42 | 9.76±0.26 | 16.94±0.58 |

The protein concentration of P-Nm2 was 2.8847 mg/mL.
